# Asthma increase among farmers: a 12-year follow-up

**DOI:** 10.3109/03009734.2010.503287

**Published:** 2011-02-11

**Authors:** Anna Rask-Andersen

**Affiliations:** Department of Medical Sciences, Occupational and Environmental Medicine, University Hospital, UppsalaSweden

**Keywords:** Agricultural Workers' Diseases, Alveolitis, allergy, asthma, Extrinsic Allergic, Farmer's Lung, farming, Longitudinal Studies, occupational disease, occupational exposure

## Abstract

Respiratory disease is a well known health hazard for farmers, but the long-term prognosis is less well known. This is a 12-year follow-up of an investigation of Swedish farmers, most of them dairy farmers. A questionnaire was mailed to all 418 farmers who were alive of the farmers originally participating in 1982. They were invited to an interview, spirometry, and blood sampling. Ninety-one per cent (380) of the farmers, 321 men and 59 women, responded to the questionnaire. The mean age was 56 years for the men and 55 years for the women. Of the group, 10% were smokers, 25% ex-smokers, and 65% had never smoked. The population estimate for asthma in the farmers was 8.9% in 1994 compared to 2% in 1982, and to 5.4%–6.6% in the general population in the region in 1982. Of the asthmatic subjects, one-third had positive RAST tests (radioallergosorbent tests). Almost 90% of the new onset asthma cases since 1982 had non-IgE-mediated asthma. Most of the IgE-mediated asthmatics had had symptoms for many years, while 70% of the non-IgE-mediated asthmatic farmers had no or only wheezing with colds 1982. Two new cases of hypersensitivity pneumonitis were identified, and 7.3% had experienced inhalation fever during the last 12 years. In general, individuals with asthma and chronic bronchitis who had left farming were in better health in 1994 as compared to 1982. In conclusion, farmers have an enhanced risk to develop asthma increasing with age. Asthma in farmers is often non-IgE-mediated.

## Introduction

Due to the large exposure to dust in their work, farmers have a higher morbidity and respiratory disease mortality than expected, and although this has been known for centuries (1) it remains a serious problem. Farmers may suffer from a number of different work-related diseases of the respiratory organs, such as asthma, chronic bronchitis, irritation of the upper respiratory tract, inhalation fever (organic dust toxic syndrome (ODTS)), and allergic alveolitis, known as farmer's lung (2–5). In a European study of animal farmers from four countries, a dose-response relationship was shown between number of hours worked daily inside confinement houses and the development of work-related shortness of breath, cough without phlegm, and inhalation fever (6). The long-term prognosis of respiratory symptoms in farmers is, however, less well known. The present study is a 12-year follow-up of an investigation carried out in 1982 of pulmonary diseases and clinical findings of Swedish farmers (7).

The purpose of the study is to investigate the long-term prognoses of the various symptoms and diseases of the respiratory organs in farmers and the way in which they are related to farm dust exposure, together with the symptoms and survey results noted 12 years ago (precipitins, RAST tests [radioallergosorbent tests], and spirometric data).

## Methods

### Study design

In 1982 a base-line study, an epidemiologic survey investigating work-related respiratory symptoms and occupational respiratory diseases, was performed among 6,267 farms (with at least one full-time farmer) in three counties of northern Sweden (7). The aim of that study was to evaluate the symptoms caused by and consequences of exposure to mould dust in farmers, such as febrile reactions and allergic alveolitis. Questionnaires on work-related and other respiratory symptoms, smoking habits, and working conditions were mailed to all farmers on these farms. The response rate was 72%. According to their answers farmers were allocated to four different groups. Only full-time farmers between 15 and 65 years of age were included (*n* = 2176), defined as a farmer working more than 30 hours per week in farming, or farming and forestry in approximately equal amounts, and who did not have any other main occupation. From each of these four groups, a stratified sample of farmers was invited to attend a thorough medical examination. Of the farmers selected, 272 farmers participated and underwent clinical examination. An additional 78 farmers were randomly selected and went through the medical examination in order to make full use of the interviewing capacity. Also a number of farmers with possible hypersensitivity pneumonitis were interviewed. Altogether 390 farmers were interviewed and examined in 1982.

For the 12-year follow-up, a questionnaire was sent in 1994 to all 418 farmers who were alive of the farmers originally selected for medical examinations in 1982. The 380 farmers who answered the questionnaire were invited to a medical examination, which included interview by a physician, blood sampling for RAST analyses and precipitins analysis, and lung function test. From the results of the questionnaire, the prevalence of asthma, work-related wheezing, chronic bronchitis, inhalation fever, and hypersensitivity pneumonitis was estimated in the whole group of farmers in the three counties. Death certificates were requested for those 35 who had died since 1982.

The study was conducted in three counties in the middle part of the Swedish region Norrland. The main production in 1982 was dairy farming (74%), often combined with forestry (33%).

### Questionnaire

The questionnaire sought to obtain information about work history, work conditions on the farm, and respiratory symptoms. The same questionnaire as 1982 was used, but some questions regarding what had happened since 1982 were added. These questions concerned items such as if the farmers were still farming, or if they had changed their farming methods. Two reminders were sent to non-responders. The asthma question was: ‘Do you have asthma?’

### Causes of death

Death certificates for those 35 farmers who had died since 1982 were requested from the Swedish Death Register.

### Clinical examination

All 380 farmers who had answered the questionnaires except for those who had moved or with serious other diseases were invited to an examination comprising spirometry, blood tests for allergy tests, precipitating antibodies, and total IgE, as well as an interview concerning symptoms, exposure, and farming methods. There were 277 (73%) who participated in the clinical examination.

### Interview

The interview was carried out using a standardized scheme by a physician specialized in occupational medicine with special experience of work-related respiratory diseases in farmers (the authour). Detailed information on symptoms and their work-relatedness was obtained. Doctor-diagnosed asthma was used as a definition of asthma.

### Allergy tests

Serum total IgE concentrations and Phadiatop were determined with the Pharmacia CAP System test (Pharmacia and Upjohn Diagnostics, Uppsala, Sweden). Phadiatop tests with serum IgE concentrations of 0.35 kU/L or more were regarded as positive. In addition, specific IgE reactivity (RAST) was measured against two storage mites (*Acarus siro* and *Lepidoglyphus destructor*) and, in farmers with asthma, against cow. Serum samples with IgE concentrations of 0.35 kU/L or more were regarded as positive.

### Precipitins

A blood sample was analysed for precipitating antibodies against a panel of 16 antigens with both an immunoelectro-osmophoresis method and the immunodiffusion method. The antigens were *Thermoactinomyces vulgaris,* *Saccharopolyspora rectivirgula (Micropolyspora faeni),* *Rhizopus,* *Cladosporium,* *Alternaria,* and *Aspergillus fumigatus.*

### Measurement of serum eosinophil markers

Whole blood was drawn in SST tubes and allowed to coagulate at room temperature for 60 min. The serum was then separated by centrifugation. Serum eosinophil cationic protein (SECP) was measured by a prototype immunofluorometric assay with the Pharmacia CAP System^™^ (Pharmacia & Upjohn Diagnostics, Uppsala, Sweden) and expressed in μg/L.

### Pulmonary function tests

Measurements of forced expiratory volume in 1 second (FEV_1_), forced vital capacity (FVC), vital capacity (VC), FEV% (FEV_1_/VC × 100), and peak expiratory flow rate (PEF) were conducted by an experienced nurse using a wedge-type spirometer (Vitalograph Ltd., Buckingham, United Kingdom). The spirometer was calibrated according to the manufacturer's instructions at least once daily. The test was conducted with the subject in an upright position and two to three slow vital capacity manoeuvres were followed by three forced expirations. The test that yielded the highest value was accepted. Standardized reference values according to Hedenström and Malmberg were used (8,9).

### Diagnosis

After the examinations, a classification of diagnoses such as inhalation fever, allergic alveolitis, work-related wheeze, doctor-diagnosed asthma, and chronic bronchitis was made by two independent physicians based on all the information obtained in the questionnaires and the interviews, but not on the results of the laboratory findings and pulmonary function tests. Medical records were obtained for those farmers who had seen a doctor because of respiratory symptoms.

### Statistical analyses

The answers to the questionnaires and the results of the tests were entered in a database, and the program SPSS was used for statistical analysis. Student's *t* test or one-way ANOVA (analysis of variance) was used to test differences in continuous variables between groups. Proportions were tested with chi-square test or Fisher's exact test (in case the expected frequency for any cell is less than 5).

### Ethical considerations

The Ethics Committee of the Uppsala University, Sweden approved the study.

## Results

### Questionnaires

A total of 91% (380) of the farmers, 321 men and 59 women, responded to the questionnaire. The mean age was 56 years (SD 12) for the men and 55 years (SD 10) for the women. Of the men, 11% were smokers, 30% ex-smokers, and 59% non-smokers. Among the women, 15% were smokers, 18% ex-smokers, and 66% were non-smokers. Difference between men and women was not significant.

Sixty-seven per cent of the farmers were still farming; 4% worked mainly in forestry, 11% had other occupations, and 18% did not work at all. Of those 89 over the age of 65 and thus on a regular state pension in Sweden, 57% were still working in farming or forestry, and 42% were working more than 30 hours a week.

Of the farms where the farmer still was farming, 61% were dairy farms, 25% had meat production, 7% were swine farmers, 3% sheep, and 2% poultry. Still, only 16% had grain production as their main production; grain was handled on 79% of the farms and hay on 91%. Potato crop was the main production on 6% of the farms. On 43% of the farms forestry was the main production.

### Population estimates

**Figure 1.**
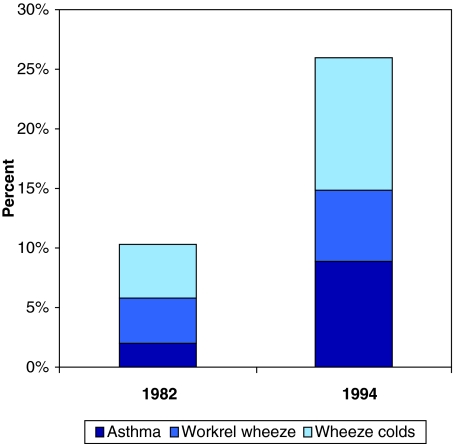
Obstructive symptoms population estimates in 1982 and 1994.

The population prevalence estimates for respiratory symptoms in the whole group of farmers in the three counties, based on the answers of the questionnaire, were as follows: of asthma 8.9%, of work-related wheeze 6.0%, of wheeze with cold 11.1%, of chronic bronchitis 7.7%, and of inhalation fever 8.1%. Compared to 1982, the prevalence of all obstructive airway symptoms had increased, especially the work-related wheeze and asthma (Figure 1). The prevalence of chronic bronchitis had increased from 3% in 1982 to 13.3% in 1994. A calculation of population estimates on the full-time farmers, only, did not change the estimates significantly.

**Figure 2.**
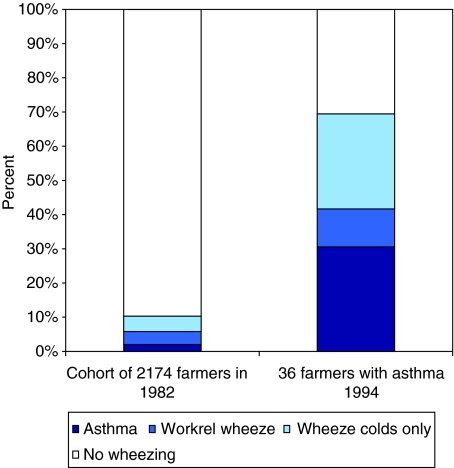
Obstructive symptoms population estimates in the whole cohort in 1982 compared to the obstructive symptoms in 1982 in the 36 farmers with asthma 1994.

Of the 36 farmers with asthma in 1994, the highest risk for asthma was found among those who already had some kind of obstructive symptoms in 1982 (Figure 2). One-third of the farmers with asthma in 1994 already had asthma in 1982, 12% had work-related wheeze, 30% had wheeze with colds, and one-third had no obstructive respiratory symptoms in 1982.

**Figure 3.**
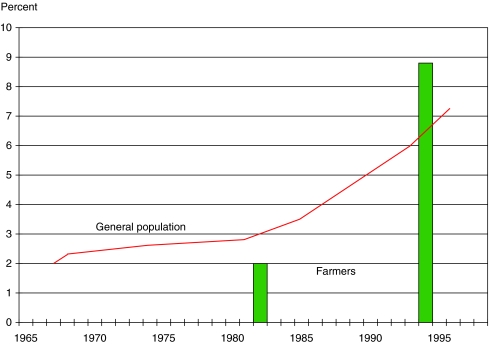
Prevalence of asthma in farmers compared to the general population.

In Figure 3 the population estimates for asthma prevalence are compared to asthma prevalence of the general population in Sweden in studies from other investigators (10,11). As shown in Figure 3, the prevalence of asthma in farmers was lower than in the general population in 1982, but higher in the same farmers in 1994.

Among the 380 farmers that answered the questionnaire, there were two new cases of hypersensitivity pneumonitis. The yearly incidence of hypersensitivity pneumonitis in 1994 is estimated to be in the same range as in 1982, i.e. 2–3/10,000 farmers.

### Causes of death

Among the 35 (30 men and 5 women) who had died since 1982, the immediate cause of death was heart diseases in 16, cerebrovascular disorders in 4, cancer in 7, respiratory tract disease in 3 and other causes in 5. Pulmonary fibrosis was the cause of death in two of the three cases who had died of respiratory disease. Asthma was a contributing cause of death in one of the deaths in heart disease and in another death from epilepsy. In total, respiratory tract disease was either immediate or contributing cause of death in five cases. Of those five, two of the subjects had pulmonary fibrosis, two had asthma, and one had other lung disease. They were all men except for one woman who died of pulmonary fibrosis.

### Clinical examinations

A total of 276 farmers, 237 men and 39 women, attended the interviews and medical examinations, but blood samples were only obtained from 270. The mean age was 56 years (SD 12) in the men and 55 (SD 9) years in the women. Of the men, 8% were smokers, 31% ex-smokers, and 60% were non-smokers. Significantly more women were smokers, 18% (*P* < 0.05). Ten per cent of the women were ex-smokers, and 72% were non-smokers.

### Interview

There were 36 cases of asthma, 27 cases of chronic bronchitis (and no asthma), 29 cases of inhalation fever (organic dust toxic syndrome (ODTS)), and 177 who had no respiratory symptoms whatsoever. The remainder (67 subjects) had respiratory symptoms that could not be categorized in any of the mentioned entities. There were significantly more smokers among the farmers with chronic bronchitis compared to the other diagnostic groups (*P* < 0.05, Table I).

**Table I.** Characteristics of the interviewed farmers in diagnostic groups.

Diagnosis		Doctor-diagnosed asthma		Chronic bronchitis		Inhalation fever (ODTS)		No respiratory symptoms		All	
Number	*n*	36		27		29		117		270	
Sex	Women/Men	7/36	19%	7/27	26%	1/29	3%	16/117	14%	36/270	13%
Age years	mean (SD)	59	±11	56	±10	55	±11	55	±11	56	±11
Smokers	*n*, %	3	6%	6^a^	22%	2	7%	13	11%	26	10%
Ex-smokers	*n*, %	14	44%	6	19%	9	30%	26	22%	74	27%
Never smokers	*n*, %	19	50%	15	59%	18	63%	78	67%	170	63%

^a^*P* < 0.01. ODTS = Organic Dust Toxic Syndrome.

### Pulmonary function test

Pulmonary function values decreased more than expected between 1982 and 1994 (Table II). The mean predicted FEV_1_ was 100% in the whole group in 1982 and had decreased more than expected to 94% predicted in 1994 (*P* < 0.01). Vital capacity (VC) also decreased more than expected: from 97% predicted value in 1982 to 92% predicted in 1994 (*P* < 0.01). Both FEV_1_ and VC were significantly lower in the symptomatic farmers (asthma, chronic bronchitis, and inhalation fever) compared to non-symptomatic farmers.

**Table II.** Results of the pulmonary function test in different diagnostic groups.

Diagnosis		Doctor-diagnosed asthma		Chronic bronchitis		Inhalation fever (ODTS)		No respiratory symptoms		All	
FEV_1_, %, predicted 1982	mean (SD)	93^b^	±15	98	±15	100	±9	104	±14	100^b^	±15
FEV_1_, %, predicted 1994	mean (SD)	82^c^	±21	91^b^	±19	93	±13	98	±15	94^b^	±17
FEV_1_ loss 1982–1994, %, of predicted value	mean (SD)	11^a^	±16	8	±9	7	±8	5	±8	6	11
FEV_1_ loss 1982–1994 mean annual losses, mL; Men	mean (SD)	60^a^	±49	53	±24	47	±31	45	±27	47	±32
FVC, %, predicted 1982	mean (SD)	94	±12	95	±16	93^a^	±9	100	±14	97^b^	±13
FVC, %, predicted 1994	mean (SD)	85^b^	±13	90^a^	±16	89^a^	±11	95	±14	92^b^	±14
FVC loss 1982–1994, %, of predicted value	mean (SD)	8	±12	4	±6	5	±6	5	±6	5	±8
FVC loss 1982–1994, mean annual losses, mL; Men	mean (SD)	59^a^	±48	40	±29	37	±36	43	±30	44	±34

^a^*P* < 0.05. ^b^*P* < 0.01. ^c^*P* < 0.001. FEV1 = Forced Expiratory Volume in 1 second; FVC = Forced Expiratory Volume in 1 second; ODTS = Organic Dust Toxic Syndrome.

A total of 7 cases of emphysema were found among the 276 interviewed farmers. Six were men and one was a woman, who was an ex-smoker. Two of the men were non-smokers, one was still smoking, and the remaining three had quit smoking.

### Allergy tests

Farmers with doctor-diagnosed asthma had significantly higher prevalence of positive allergy tests as well as significantly higher levels of total IgE compared to the other diagnostic groups (Table III). In the asthma group, 28% (10/36) had positive Phadiatop and/or positive RAST towards storage mites and/or cow dandruff. The farmers with inhalation fever had a higher prevalence of positive precipitins, and farmers with asthma and chronic bronchitis had a trend to lower prevalence compared to the non-symptomatic farmers, although the differences were not significant.

**Table III.** Results of allergy and precipitins tests in different diagnostic groups.

Diagnosis		Doctor-diagnosed asthma		Chronic bronchitis		Inhalation fever (ODTS)		No respiratory symptoms		All	
Phadiatop +	*n*, %	5^a^	14%	3	11%	3	10%	4	3%	18	7%
RAST storage mites +	*n*, %	3	8%	0	0%	3	10%	3	3%	7	3%
Phadiatop + or RAST storage mites +	*n*, %	8^b^	23%	3	11%	4	13%	6	5%	25	9%
RAST cow dander +	*n*, %	3	8%								
Allergy tests + (Phadiatop+ or any RAST+)	*n*, %	10	28%								
Total IgE	mean (SD)	147^c^	±239	40	±39	63	±115	48	±77	60	±114
ECP mg/L	mean (SD)	10.9	±6.0	11.0	±6.7	8.6	±4.8	9.6	±5.4	10.0	±5.6
Precipitins(+), +, and ++	*n*, %	4	11%	2	7%	8	27%	23	20%	48	18%

^a^*P* < 0.05. ^b^*P* < 0.01. ^c^*P* < 0.001. ODTS = Organic Dust Toxic Syndrome; ECP = Eosinophil cationic protein.

**Table IV.** IgE-mediated asthma compared to non-IgE-mediated asthma.

	Doctor-diagnosed asthma 1994	
Obstructive symptoms 1982	Allergy test positive 1994	Allergy test negative 1994
No symptoms	1	10
Wheeze colds	1	9
Definite wheeze	0	4
Asthma	6	4
Total	8	27

Of eight cases (two cow dandruff-positive ones not included) with IgE-mediated asthma in 1994, 75% (6/8) had asthma in 1982 (Table IV). Only one of the eight cases of IgE-mediated asthma was completely without respiratory symptoms in 1982, and one had wheezing but only on colds. In contrast, of the 27 non-IgE-mediated asthma cases, only 15% (4/27) had asthma in 1982. Thirty-seven per cent (10/27) had no respiratory symptoms whatsoever in 1982, and 33% (9/27) had wheezing but only with colds. Fifteen per cent of the non-IgE-mediated asthma cases (4/27) had work-related wheezing in 1982. Thus, most of the IgE-mediated asthmatics had experienced symptoms for many years, while 70% of the non-IgE-mediated asthmatic farmers had none or only wheezing with colds in 1982. Most of the new onset asthma cases had non-IgE-mediated asthma (23/27 = 85%).

The farmers with non-IgE-mediated asthma had significantly lower FEV_1_ and VC than the IgE-mediated asthma cases: FEV_1_ 76% of predicted versus 97% of predicted, *P* < 0.05; and VC 82% of predicted versus 96% of predicted, *P* < 0.01. Farmers who were still farming and were exposed to farm dust had a trend to lower FEV_1_ (79% of predicted) compared to farmers who had quit farming (91% predicted), although this difference was not significant. Vital capacity was almost the same in farmers who farmed compared to farmers who had quit farming, 84% of predicted versus 88% of predicted. Eosinophil cationic protein (ECP) was 12.0 μg/L in the farmers with IgE-mediated asthma compared to 10.6 μg/L in the farmers with non-IgE-mediated asthma, but the ECP values did not correlate with the spirometry values or exposure to farm dust.

### Exposure

During the interviews, many farmers told us that their obstructive symptoms had improved since their exposure decreased, when they left dairy farming for beef farming or completely stopped farming. Several farmers had been taking asthma medication that they were able to end after the exposure decreased. Also, two farmers who had chronic bronchitis for decades had major improvement after being admitted to hospital for some months after work accidents involving fractures.

Several of the farmers with work-related respiratory symptoms had also tried to reduce their dust exposure by going over to silage instead of hay, using tower silos or baled silage. Others had installed automatic feeding. The farmers could often describe what worsened their airway symptoms such as dust from hay or grinding of grain. One farmer, allergic to pollen, tried to harvest the hay as early as possible to avoid worsening of symptoms during the hay harvest. Some had stopped with grain completely because of bad revenues and as a side-effect noted that their airway symptoms improved.

In 1982, the method of silage in plastic bales had not been introduced in Sweden, but they were introduced prior to 1994, with a decrease of haymaking. In the northern part of the region investigated, the climate is so cold that it is hard to make grain farming profitable. Instead silage was raised. In addition, the cattle were fed with purchased pellet fodder so the cattle got all the nutrients needed.

### Hypersensitivity pneumonitis

Two new cases of hypersensitivity pneumonitis were identified. The first case, a 50-year-old woman, fell ill in December 1982, and the cause was mouldy straw. She had a complete recovery. The other case was a 42-year-old man affected in 1984, and the cause was mouldy wood chips for the purpose of heating his dwelling. He had inhalation fever after handling mouldy wood chips earlier, in the 1970s. This time he slowly got progressive dyspnoea. As he did not realize that the cause was the mouldy wood chips, he continued to use the wood chips. He went to his family doctor and received several treatments with different antibiotics. Finally, he went to the emergency room and was admitted to intensive care. By then, he had started to suspect the mouldy wood chips, and informed his doctor. Dramatically, an arterial blood sample was drawn and the farmer noticed that the blood was blue. PaO_2_ was 6.2 kPa. The farmer stopped using wood chips. He had a complete recovery.

## Discussion

In this longitudinal study of respiratory diseases in farmers with a follow-up time of 12 years, asthma prevalence had increased from 2% to 8.9%. That is higher than the asthma prevalence in the general population in the same region in the same age group (50–60 years) investigated with similar techniques during the same time period (12). By contrast, the asthma prevalence in 1982 when the mean age of our farmers was 44 years was lower than that of the general population. There is an increase in asthma in the whole Western world, including Sweden, but the increase of prevalence of asthma in the farmers of our study increased more than expected showing that farmers have an enhanced risk to develop asthma increasing with age.

Almost 90% of the new onset asthma cases developing during the follow-up time had non-IgE-mediated asthma. Positive RAST tests were only found in 28% of the asthma cases, although a RAST test specific for farming such as storage mites and cow dandruff was used in addition to a RAST panel of common inhalation allergens (Phadiatop). Farmers with IgE-mediated asthma had higher ECP values than farmers with non-IgE-mediated asthma (*P* < 0.001) in accordance with findings of a Norwegian study (13). In that study, ECP showed significant associations with airway obstruction and numbers of RAST allergen-positive atopic asthmatics, but no such trends could be seen in this study.

Work-relatedness of respiratory disorders is hard to evaluate in farmers, since they are working and living in the same environment. Farmers often do not have weekends off or vacations, so they might not know if the exposures at work worsen their symptoms. However, in this study even senior farmers over the age of 65 years were included. They were on a pension, and many had reduced their activity in farming. Thus, the dust exposure was often lower in the senior farmers in 1994 compared to 1982. Results of the interviews revealed work-relatedness, with deterioration occurring in conjunction with exposure to dust at work in many farmers, and an improvement was noted in those who had reduced dust levels or stopped working as farmers.

Pulmonary function values had decreased more than expected between 1982 and 1994 even in non-symptomatic farmers with 67% non-smokers. Especially large declines were seen in the farmers with asthma and chronic bronchitis. Several earlier studies have demonstrated that farmers more often have a reduced FEV_1_ and a more rapid decline in lung function than controls (14–16). The mean annual losses in FEV_1_ and FVC were in the same order of magnitude as for workers exposed for 20 years or more in the Canadian grain industry (17).

Cigarette smoking is the leading cause of Chronic Obstructive Pulmonary Disease (COPD) and chronic bronchitis. As expected, there were significantly more smokers among the farmers with chronic bronchitis compared to the other diagnostic groups, but emphysema was found in two never-smoking farmers. Since emphysema is uncommon in non-smokers, it is possible that the exposure to farm dust contributed to the emphysema development. In a Swedish linkage of the 1960 Census and the Causes of Death Register, the only occupational group showing an increased mortality due to pulmonary emphysema after adjusting for smoking habits was the occupational group containing farmers as its largest subgroup (18). Exposure to vapours, gases, dusts, or fumes in the work-place during the longest-held job was associated with a 2-fold increase in risk of COPD (OR 2.0; 95% CI 1.6–2.5) in an American study (19). In a recent study by the same group it was found that work-place exposures were strongly associated with an increased risk of COPD (20). Joint exposure to both smoking and occupational factors markedly increased the risk of COPD (OR 14.1; 95% CI 9.33–21.2). An especially elevated risk for chronic bronchitis in non-smokers has been reported in French dairy farmers (21).

As in 1982, inhalation fever and chronic bronchitis are still very common among farmers, whereas hypersensitivity pneumonitis is a rare disease. Of the 35 deaths since 1982, a respiratory tract disorder was either immediate or contributing cause of death in 5 cases. Of those five, two of the subjects had pulmonary fibrosis, two had asthma, and one had other lung disease as immediate or contributing cause of death.

Questionnaires to diagnose asthma have been developed and validated (10), but these were not available in 1982. Accordingly, therefore we preferred to use the same question as in 1982 (‘Do you have asthma?’) to ascertain asthma in the questionnaire. It is certain that there was an increase of symptoms that the farmers or their doctors would call asthma, but it is less certain whether all these farmers would have asthma if strictly diagnosed. It would have been useful with a methacholine test to confirm the diagnosis of asthma, but it was not possible within the scope of this study. The definition of ‘doctor-diagnosed asthma’ was used to classify cases in diagnostic categories after the interviews to compare results of pulmonary function test and allergy tests. Since we found a tendency that more farmers with symptoms came to the clinical examinations compared to asymptomatic farmers, and because more farmers had answered the questionnaire than those attending the interviews, we chose to use the questionnaire data for the population estimates. One of the reasons for not attending the interviews was that farmers had moved and could not be present at the test centres. Other reasons were high age or other serious disease.

It is well established that farmers may suffer from a number of different work-related diseases of the respiratory organs (2,5), and asthma was described in 1924 (22). Our results are supported by a number of other studies. In a Danish study from 1988, the mean prevalence of asthma in a representative sample of 1,685 farmers was 7.7%, lowest (3.6%) among farmers aged 30–49 and highest (11.8%) among farmers aged 50–69 years (14). Logistic regression analysis revealed that age (OR 5.8; 95% CI 2.8–12.2) and pig farming (OR 2.0; 95% CI 2.0–3.5) were risk factors for self-reported asthma. The prevalence of asthma among farmers and aged matched subjects from the general population was the same in the age group 30–49 years, but significantly higher among farmers aged 50–69 compared with aged matched subjects from the same sample (OR 2.25; *P* < 0.001).

Also, in a random sample of 7,496 European farmers from Denmark, northern Germany, Switzerland, and Spain, all symptoms related to asthma (wheezing, shortness of breath, asthma attacks) and nasal allergies had significantly lower prevalences in farmers than in the general population (6). The prevalence of asthma (2.8%; 95% CI 2.4–3.2 in farmers of all age groups) was significantly (*P* = 0.001) lower in farmers aged 20–44 years (1.3%; 95% CI 0.9–1.7) compared to the prevalence in an age-matched sample of the general European population (European Community Respiratory Health Survey (ECRHS)) (3.2%; 95% CI 2.9–3.9) (6). In crop farmers in the same European countries, 3.2% reported asthma, which was similar to the prevalences found for the general European population and did not suggest a higher prevalence of symptoms of obstructive lung disease among these European farmers (23). No analysis on different age groups of farmers was presented in this study. The analysis of specific crops showed a high risk of bronchial asthma (OR 2.1; 95% CI 1.1–3.9) in workers who grew flowers after adjustment for covariates.

In a study of farmers in central Sweden, the prevalence of doctor-diagnosed asthma was significantly lower (*P* < 0.001) in farmers below the age of 50 years, 1.9%, compared to 7.2% in the general population, but 9.4% in farmers aged 55–65 years (24). Significantly increased mortality from asthma was found among male farmers in a study of Swedish official mortality statistics (25). In another Swedish study, an increased risk of hospitalization for asthma was found among male farmers (26).

In a Swedish study in Gotland, an island in the Baltic Sea, the prevalence of asthma had increased significantly during the previous 12 years (5.3% versus 9.8%) as well as the prevalence of asthma among atopic subjects (3.1% versus 4.9%) (16,27). In this study, 461 dairy farmers were investigated in 1996, and 65 (14.1%) of these subjects participated in the study in 1984. As in our study, a number of asymptomatic farmers (6/12) in 1984 had developed respiratory symptoms, and 19/33, who in 1984 had rhinoconjunctivitis only, were reporting symptoms from the lower airways as well. In contrast to the findings in the present study, these findings could not be explained by a shift towards an older and more symptomatic population, since the mean ages were almost the same in 1984 and 1996. The follow-up in Gotland was a cross-sectional study not including retired farmers and people that had left farming as in the present study.

In an analysis of data from the European Community Respiratory Health Survey (ECRHS) in ten European countries as well as USA and New Zealand, the highest risk of asthma (defined as bronchial hyperresponsiveness and reported asthma symptoms or medication) attributed to occupation was found for farmers, OR 2.62 (95% CI 1.29–5.35) (28). Results for farmers were consistent between 12 countries including USA and New Zealand participating in the survey.

In France, the prevalence rates of asthma were particularly high in former farm workers (29). Farmers appeared to have a higher risk of both cumulative (OR 2.30; 95% CI 1.00–5.47) and current asthma (OR 5.35; 95% CI 1.33–21.50) compared to white-collar workers adjusted for age, sex, and smoking history. In New Zealand, the prevalence for combination of wheeze and non-allergic airway hyperresponsiveness was significantly increased (OR 4.16; 95% CI 1.33–13.1) for farmers and farm workers (30). Data from Finnish surveillance systems for occupational diseases show that the incidence rate of occupational asthma is exceptionally high in farmers and attributed to the custom in Finnish farming to brush the cows daily (31).

In other cross-sectional studies of farmers, the reported prevalence of asthma has varied between regions and has also varied dependent on types of farming. According to a review of Omland, healthy worker selection, heterogeneity in diagnosis, misclassification, age differences, difference in time of study, and small study populations resulting in low statistical power might also be factors explaining why no difference between farmers and the general population has been observed in some studies (2).

In a large Norwegian study, significant risk factors for current asthma were asthma heredity (OR 2.9; 95% CI 2.1–3.9), asthma as a child or adolescent (OR 22.2; 95% CI 15.2–32.4), animal production (OR 1.6; 95% CI 1.1–2.2), and age 40–69 years (OR 1.8 to 4.6; 95% CI 1.1–7.5) (32). In a Danish study of 1,901 farming students (of whom 210 were females) and 407 rural controls, female sex (OR (males) 0.5; 95% CI 0.3–0.8), asthma in the family (OR 1.6–3.4), and smoking (OR 1.7; 95% CI 1.2–2.4) were factors significantly associated to asthma (33). The prevalence of asthma-like symptoms was between 5.4% and 21%, but no difference of the prevalence of asthma-like symptoms was observed between farming students and controls.

Exposure to chemicals is another risk factor for asthma. In a Canadian study, exposure to carbamate insecticides was shown to increase the risk for asthma (OR 1.8; 95% CI 1.1–3.1) (34). In pig farmers, the use of disinfectants (quaternary ammonium compounds) (OR 9.4; 95% CI 1.6–57.2) and aspects of disinfecting procedure were associated with the prevalence of asthma (35).

There are at least three kinds of selections in action influencing the prevalence of asthma in farmers. First, farmers' sons (preferably) inherit the farm as well as the occupation. Since children raised on a farm also have a decreased prevalence of atopic diseases (36), the asthma prevalence of young people starting out farming is low.

Second, the few farm children that have asthma and allergy are not prone to take up farming as farm children start helping out on the farm at an early age and will soon realize that farming is not a suitable occupation for somebody with atopy and allergy towards e.g. cat, dust mites, or pollen. All the common inhalative allergens are found on farms—pollens, animal dandruff, moulds, and mites (37). The healthy worker effect, ‘selection in’, is thus probably particularly strong in farming due to self-selection already before having actually started the job. Actually, during our interviews a number of farmers gave comments such as: ‘My son can’t take over the farm because he has asthma'. Possibly, there is a long-term healthy non-allergic workers effect over multiple generations (6).

Third, farmers that get asthma might leave farming because of work aggravation and get another job—survivor bias, that is ‘selection out’ from the occupation (38,39). This might be particularly true for younger farmers that have the opportunity to get another job perhaps through retraining. For an older farmer that develops asthma it might be impossible to get another job, especially if he lives in a remote area with few job opportunities. Studies from both Sweden and Finland have showed that farmers have a lower risk of leaving their occupation than do people in other lines of work, but farmers with allergies more often changed occupation than did farmers with other diseases (38,40). Farming is not only a way to make a living; it is a life-style. Farms pass from generation to generation. A farmer with asthma might not want to leave the farm. Studies have shown that farmers retire early less often than those in other occupations (38). Therefore farmers try to continue farming until retirement maybe by slightly changed production such as changing from dairy farming to the less dusty beef farming, using silage, or using a respiratory device. These are reasons that might explain the high asthma prevalence of older farmers and the low asthma prevalence in cross-sectional studies of younger farmers as illustrated by our study and others.

The lower asthma prevalence in farmers found in a number of studies can be explained by such selection mechanisms. For example, in a Dutch study of swine farmers, the same prevalence of asthma was found as in rural controls, but atopy and symptoms of allergy during childhood were less prevalent in the pig farmers (4.6%) compared to controls (14.6%) (35). In a Canadian report, four female swine workers consulted a chest physician because of an acute onset of wheezing and cough suggestive of asthma within weeks of commencing full-time employment in intensive swine production facilities (41). All four had quit working in swine production illustrating selection mechanisms that partly explain why the asthma prevalence in farmers may be lower than in the general population. Three of these cases fulfilled the diagnostic criteria of asthma consistent with occupational asthma according to American College of Chest Physicians Guidelines (42). The fourth case had normal spirometry and bronchial responsiveness; however, she experienced severe symptoms, requiring medication, and methacholine challenge was conducted several months after exposure cessation. Two of the workers were atopics; one had border-line atopy on a skin prick test.

Usually in studies of occupational diseases, only subjects in the work-force are included, with an age ranging from the upper teens to 65 years (depending on the retirement age in the country). In this study, even farmers over the retirement age limit were included, which rendered important information. One-fourth of the older farmers were still working full time, while the others either farmed less intensively or had completely left farming, and the exposure to farm dust had decreased. During the interviews, it was obvious that farmers with both asthma and chronic bronchitis had improved after reduction of exposure. Several farmers that had been on continuous asthma medication had been able to stop medication after quitting farming. Changes in exposure level probably explain the lower asthma prevalence in farmers older than 65 years (the retirement age in Sweden) compared to farmers aged 55–65 found in another Swedish study (24).

In our study, positive RAST tests were only found in 28% of the farmers with asthma although RAST tests specific for farming such as storage mites and cow dandruff were used in addition to a RAST panel of common inhalation allergens (Phadiatop). Also, only 15% (4/27) of the new onset asthma cases since 1982 had IgE-mediated asthma. The prevalence of sensitization to storage mites is much lower in the present study compared to studies in Gotland carried out in 1983 and 1996, when about 6% of all the farmers and 20%–25% of the asthmatic farmers had allergy to storage mites (16,27). One explanation might be that Gotland is an island in the middle of the Baltic Sea with a milder, more humid climate during the winter compared to the northern parts of Sweden in this study, where storage mites probably would freeze to death during cold winters in a drier climate.

In the Finnish surveillance data, cow dandruff was the most common of the primary causative animal epithelia and barley the most common of the primary causative flours in farmers (31). In Finland, farm-women take care of cows, and animal epithelia were more commonly noticed as the primary cause in female farmers (77%) than in male farmers (62%). The high rate of allergy to cow dandruff in Finnish farmers has been explained by the Finnish habit of brushing the cows daily. In our study, only 8% of the farmers with asthma had a positive RAST to cow dandruff. Among Swedish farmers, cow dandruff seems to be of less importance as an occupational allergen, although cow was the most common cause of sensitization to animal dandruff (4.2%) in the Swedish study on Gotland (16). The prevalence of brushing cows comparing Swedish and Finnish farmers has not been systematically studied.

It is not surprising that farmers do develop asthma because of the high exposure to farm dust containing both a number of well known allergens as well as airway irritants. Also, there are studies that have shown that perfectly healthy farmers on farms without any mould problems have an increased number of inflammatory cells in their bronchioalveolar lavage (43). The same research group has also shown an increasing number of positive methacholine tests in naive subjects after exposure in swine confinement buildings (44). What is surprising is the paradox that growing up in these kinds of environment decreases the risk of atopic diseases. The protective effect of contact with live-stock and poultry is consistent in several studies (36).

In this study, the risk to develop asthma was largest in the farmers with any kind of respiratory symptom since 12 years. Even milder respiratory symptoms that do not fit the diagnostic criteria of a specific disease entity, such as work-related wheeze or wheezing with colds, have to be taken seriously. This is supported by other studies of farmers. Acute symptoms during work predicted chronic bronchitis in French farmers (45). European animal farmers with nasal irritation during work had a four times increased risk of bringing up phlegm in the winter time (6). Studies of occupational asthma in other occupational settings have also shown that the higher the exposure, the greater the risk for developing occupational asthma, and, by implication, lowering the level of exposure reduces the incidence of disease (39). It can be concluded from large well designed studies in different countries using standardized questionnaires, the same sampling methods, and standardized spirometric measurements that there is a significant relationship between bad working conditions (such a bad ventilation or high respirable dust concentrations) and work-related respiratory symptoms (6,23,46).

In conclusion, the finding in this study shows that farmers have an increased risk for adult onset asthma, often not IgE-mediated, probably attributable to occupational dust exposure. When discussing how high the risk is, one must take into account that young farmers have a low asthma prevalence due to selection and to the fact that most of them have been raised on farms resulting in a lower prevalence of asthma compared to people that have grown up in other environments. However, after decades of exposure to high levels of dust containing multiple agents (bacteria, moulds, endotoxin, allergens, ammonia, and other irritant gases) that may act in an additive or synergistic manner, the result is a high prevalence of respiratory disease even in such an initially healthy population as farmers. For the affected farmers asthma implicates serious problems with decreased life quality due to aggravation of asthma symptoms caused by daily exposure to farm dust in a disease that can be fatal. It was evident, in this study, that after farmers had improved their working conditions or quit farming their asthma improved and even disappeared completely in some farmers. Farmers with even mild respiratory symptoms belong to a risk group with probable high exposure to dust. These farmers warrant concern, should be monitored, and should receive advice on how to minimize exposure in their farms preferably by improving farming methods and/or by using respirators. Smoking farmers are also a risk group and should receive advice and help to quit smoking.
